# Long noncoding RNA HOXB13‐AS1 regulates HOXB13 gene methylation by interacting with EZH2 in glioma

**DOI:** 10.1002/cam4.1718

**Published:** 2018-08-13

**Authors:** Yu Xiong, Wei Kuang, Shigang Lu, Hua Guo, Miaojing Wu, Minhua Ye, Lei Wu

**Affiliations:** ^1^ Department of Ophthalmology The Second Affiliated Hospital of Nanchang University Nanchang China; ^2^ Department of Neurosurgery The Second Affiliated Hospital of Nanchang University Nanchang China

**Keywords:** epigenetic, glioma, HOXB13‐AS1, long noncoding RNAs, methylation

## Abstract

Dysregulation of long noncoding RNAs (lncRNAs) has been implicated in human diseases, in particular, cancers. In this study, we determined the expression of an lncRNA, HOXB13‐AS1, involving in glioma. We showed that HOXB13‐AS1 was significantly upregulated in glioma tissues and cells and was negatively correlated with its surrounding gene HOXB13 levels. Functional experiments in vitro and in vivo revealed that high level of HOXB13‐AS1 increased cell proliferation and tumor growth by promoting cell cycle progression. Conversely, knockdown of HOXB13‐AS1 resulted in decreased cell proliferation and tumor growth. Mechanistically, we showed that HOXB13‐AS1 overexpression increased DNMT3B‐mediated methylation of adjacent gene HOXB13 promoter by binding with the enhancer of zeste homolog 2 (EZH2) using bisulfite sequencing PCR (BSP), epigenetically suppressing HOXB13 expression. Additionally, the interaction between HOXB13‐AS1 and HOXB13 was validated by RNA immunoprecipitation (RIP) and chromatin immunoprecipitation (ChIP) assays using antibody against to EZH2. Taken together, our study indicated that HOXB13‐AS1 could regulate HOXB13 gene expression by methylation HOXB13 promoter and acts as an epigenetic oncogenic in glioma.

## 1 INTRODUCTION

Glioma is one of the most leading cause of central nervous system (CNS) tumors death worldwide^1^, ^2^ and is increasing rapidly especially. As we all known, glioma is a complicated disease involving multistep progression and genetic or epigenetic abnormalities, including mutations of genes, and genomic methylation patterns of specific tumor suppressor genes (TSG) or oncogenes.^3^, ^4^, ^5^, ^6^ Therefore, a deeper understanding of the molecular mechanisms involved in glioma tumorigenesis is fully urgent for the improvement of glioma diagnosis and treatment.

Recently, with the advanced development of large‐scale technology, a growing number of observations have determined that numerous noncoding sequences occupy more than 90% of the human genome.^7^ Long noncoding RNAs (lncRNAs), as a class of the noncoding RNA molecules, are greater than 200 nucleotides in length with limited protein coding capacity.^8^, ^9^ Up to date, a number of lncRNAs have been received increased attention to participate in multiple major biological processes, including cellular homeostasis, development, differentiation, pluripotency of embryonic stem cells, and genomic imprinting.^10^, ^11^, ^12^ In addition, lots of evidence highlight that numbers of lncRNAs are well documented to be associated with human diseases and carcinogenesis, such as HOTAIR in hepatocellular carcinoma,^13^ lung cancer,^14^ breast cancer,^15^ and glioma.^16^, ^17^, ^18^ In fact, the aberrant level of lncRNAs has been well recognized as biomarkers for future cancer diagnostic, therapeutic, or prognostic.^19^, ^20^

The underlying molecular mechanisms used by lncRNAs in intertwined gene regulatory network are complex and diverse.^21^ A growing list of abnormal expression of lncRNAs was validated to participate in the regulation of gene expression by multiple ways, including transcriptional/post‐transcriptional processing, chromatin remodeling, RNA decay, and epigenetic modification via acting as decoys, guides, or scaffolds.^22^ In cancer, lncRNAs could act as a modular scaffold through the recruitment of PRC2 (polycomb repressive complex 2), which modulate silencing marks at targeted genes and promote tumor progression.^23^ Additionally, some of lncRNAs control the post‐transcriptional mRNA processing through complementary base‐pairing interactions with the target mRNA, which inhibit RNases to degrade the mRNA.^24^ The another mechanisms of lncRNAs in which action to regulate critical cellular processes are via serving as competing endogenous RNAs (ceRNAs), negatively regulating microRNAs.^25^

HOXB13‐AS1 is a 564 nucleotide‐lncRNA and situates in the homeobox (HOX) gene locus on chromosome 17, which has been identified to function critical roles in embryo development and numerous cellular processes.^26^, ^27^, ^28^, ^29^ A substantial body of scientific evidence reported that aberrations in HOX gene expression associated with many different types of malignancies. Recently, certain kinds of lncRNAs have been reported to be located on HOX gene clusters, including HOXB13‐AS1. Subsequent studies showed that HOXB13‐AS1 was highly expressed in human prostate, uterus, distal colon, and stomach tissues.^30^, ^31^ Moreover, another study also reported that HOXB13‐AS1 promoted breast cancer initiation and progression by activating PI3K‐AKT‐mTOR pathway.^32^ However, the expression and biological roles of HOXB13‐AS1 in glioma have not been fully characterized. Because of the higher expression and function roles of HOXB13‐AS1 in human cancer attracted us to choose HOXB13‐AS1 as the focus to investigate in this study, we thereby considered whether the overexpression of HOXB13‐AS1 gene was associated with glioma tumorigenesis.

In the report, we determined that HOXB13‐AS1 was significantly overexpressed in glioma tissues and cells. Moreover, we demonstrated that HOXB13‐AS1 could contribute to glioma cell proliferation by binding with the enhancer of zeste homolog 2 (EZH2), epigenetically suppressing its neighbor gene HOXB13 expression.

## 2 MATERIALS AND METHODS

### 2.1 Tissues collection

Ninety‐six of glioma tissue samples were obtained from patients who were diagnosed with glioma. The glioma patients were pathologically diagnosed, and surgical resection of glioma was carried out at The Second Affiliated Hospital of Nanchang University (Nanchang, China). Normal brain tissues (NBT) were collected from the patients who died in traffic accidents during the same period at The Second Affiliated Hospital of Nanchang University. None of the individuals had received any chemotherapy or radiotherapy before surgery. The use of tissues for this study was approved by the ethics committee of The Second Affiliated Hospital of Nanchang University. All patients before any operation have signed the informed consent.

### 2.2 Cell culture

Human glioma cell lines (U251, U87, SHG‐44, and SHG139) and the normal human astrocytes cell line (NHA) were purchased from Cell Bank of Chinese Academy of Sciences (Shanghai, China) and cultured in DMEM or RPMI 1640 medium containing 10% fetal bovine serum (Gibco, Carlsbad, CA, USA), at 37°C with 5% CO2. All media were additionally supplemented with 100 U/mL penicillin and 100 mg/mL streptomycin (Gibco, Invitrogen, Shanghai, China).

### 2.3 Subcellular fractionation location

Nuclear and cytosolic fractions of glioma cells were separated using the PARIS Kit (Life Technologies, Carlsbad, CA, USA). HOXB13‐AS1 expression was evaluated in the subcellular fractionation of glioma cells by real‐time PCR (qRT–PCR).

### 2.4 Plasmids’ construction and transfection

The HOXB13‐AS1 expression construct was created by cloning the full‐length fragment of HOXB13‐AS1 into the pcDNA3.1 vector. For HOXB13‐AS1 silence experiments, the siRNAs against HOXB13‐AS1 and scrambled negative control siRNA (si‐NC) were obtained from Sigma‐Aldrich (St. Louis, MO, USA). The siRNA sequences efficiently targeting HOXB13‐AS1 were designed and expressed as shRNAs: sh‐HOXB13‐AS1. Cell transfection was performed using Lipofectamine 2000 (Invitrogen, Carlsbad, CA, USA) according to the manufacturer's introductions. The transfection efficiency was evaluated by qRT‐PCR analyses.

### 2.5 Lentivirus construction and cell transduction

293T cells (5 × 10^4^) were plated in the wells of 24‐well plates. The pGV248‐shHOXB13‐AS1 or either control or lentiviral packaging plasmids were cotransfected into 293T cells following the manufacturer's protocol. After 48 hours transfection, the lentivirus partials were harvest and viral titers were determined by quantification. The lentivirus with pGV248‐shHOXB13‐AS1 was then used to infect glioma cells and was selected to obtain stable clones. The HOXB13‐AS1 downregulation in lentivirus‐transduced stable clones was further verified by RT‐qPCR. The stable expression cell lines with HOXB13‐AS1 downregulation were maintained in medium and were then used for in vivo experiments.

### 2.6 RNA extraction and qRT‐PCR analyses

Total RNA was extracted from cells or tissues using TRIzol reagent (Invitrogen), and 1 μg RNA was reverse‐transcribed to cDNA using reverse transcriptase (Promega, Shanghai, China) according to the manufacturers’ protocols. qRT‐PCR analyses were performed using the SYBR Premix Ex Taq (Takara, Dalian, China) in ABI 7500 Real‐Time PCR System as described previously.^33^ The relative expression level of target RNAs in this study was evaluated using the 2^−ΔΔCt^ method, normalized to housekeeping gene glyceraldehyde 3‐phosphate dehydrogenase (*GADPH*). The real‐time PCRs were performed in triplicate.

### 2.7 Western blotting assay

Protein lysates were prepared using RIPA buffer (Beyotime, Shanghai, China). Then, proteins were separated by a 10% sodium dodecyl sulphate‐polyacrylamide gel electrophoresis (SDS‐PAGE) and transferred to PVDF membranes (Bio‐Rad, Shanghai, China). Western blotting was performed based on a previous study.^34^ The membrane was blocked with 5% nonfat dry milk in TBS for 1 hour at room temperature and was probed with specific primary antibodies at 4°C overnight. Antibodies against HOXB13, DNMT3B, and GAPDH were purchased from Santa Cruz. The protein bands were visualized and standardized to that of GAPDH.

### 2.8 RNA binding protein immunoprecipitation (RIP) assay

RIP experiments were performed using the EZ‐Magna RIP kit (Millipore, China) following the manufacturer's guidance. Human anti‐EZH2 antibody (Abcam, Shanghai, China), SUZ12 (Santa Cruz, Shanghai, China), LSD1 (Santa Cruz), or control IgG was used. The immunoprecipitated RNA was isolated and was further subjected to qRT‐PCR analysis.

### 2.9 Chromatin immunoprecipitation (ChIP) assay

Chromatin immunoprecipitation was performed using EZ‐Magna ChIP TMA Chromatin Immunoprecipitation Kit (Millipore, Billerica, MA, USA) following the manufacturer's instructions. Formaldehyde cross‐linked chromatin obtained from glioma cells was sonicated to generate DNA fragments from 200 bp to 300 bp. The chromatin DNA was precipitated with the antibodies including EZH2 antibody (Abcam), and further analyzed by qRT‐PCR. The IgG antibody was used as control.

### 2.10 DNA extraction and bisulfite sequencing PCR (BSP)

DNA from glioma cell lines was isolated using the DNeasy^®^Blood and Tissue Kit (Qiagen, Valencia, CA, USA), bisulfate modified, and then subjected to BS analysis with the EpiTect^®^ Bisulite Kit (Qiagen) according to the manufacturer's instructions.

### 2.11 Cell viability and colony assays

For cell viability assay, glioma cells transiently transfected with pCDNA3.1‐HOXB13‐AS1 or siRNA‐HOXA‐AS1 were allowed to grow 24, 48, 72, and 96 hours and were treated with a Cell Counting Kit‐8 (Dojindo Molecular Technologies, Rockville, MD, USA), respectively, according to the manufacturer's instruction. For colony formation assay, treated and untreated glioma cells were placed at a density of 50 cells per well into 6‐well plates and then cultured at 37°C with 10% FBS media for 2 weeks. The ability of colony formation was measured by counting the number of stained colonies.

### 2.12 Cell cycle analysis

The cells transfected with pCDNA3.1‐HOXB13‐AS1 or siRNA‐HOXA‐AS1 were harvested, washed with PBS and fixed overnight in 4% formaldehyde at −20°C. DNA content was stained with 50 g/mL propidium iodide (PI) (Sigma) and then quantitated by a FACScan flow cytometry (BD Biosciences, Beckman Coulter, USA).

### 2.13 Mouse xenograft experiments

All animal‐related protocol was approved by the institutional ethics committee of Animal Experiments of Nanjing Medical University. 2 × 10^6^ cells transfected with pCDNA3.1‐HOXB13‐AS1, shRNA‐HOX13‐AS1, or empty vector (NC) were trypsinized to single‐cell suspension in 100 μL PBS and were then implanted subcutaneously in the dorsal flanking sites of 10‐week‐old BALB/c nude mice (Animal Center of the Chinese Academy of Science, Shanghai, China) for 4 weeks. The tumor growth was measured every 3 days in the HOXB13‐AS1‐overexpression group, HOX13‐AS1‐downregulation group, and the NC group. Tumor volumes were determined as length × width^2^ × 0.5

### 2.14 Statistical analysis

All experimental data were performed using SPSS version 18.0 (SPSS Inc., Chicago, IL, USA). One‐way analysis of variance (ANOVA) was used to analyze for differences between multiple groups. The chi‐square test or Student's *t* test was used to examine the differences between two groups. Data are expressed as mean ± SD. A value of *P *<* *0.05 was chosen for statistical significance.

## 3 RESULTS

### 3.1 The expression levels of HOXB13‐AS1 in glioma tissues and cell lines

**Figure 1:**
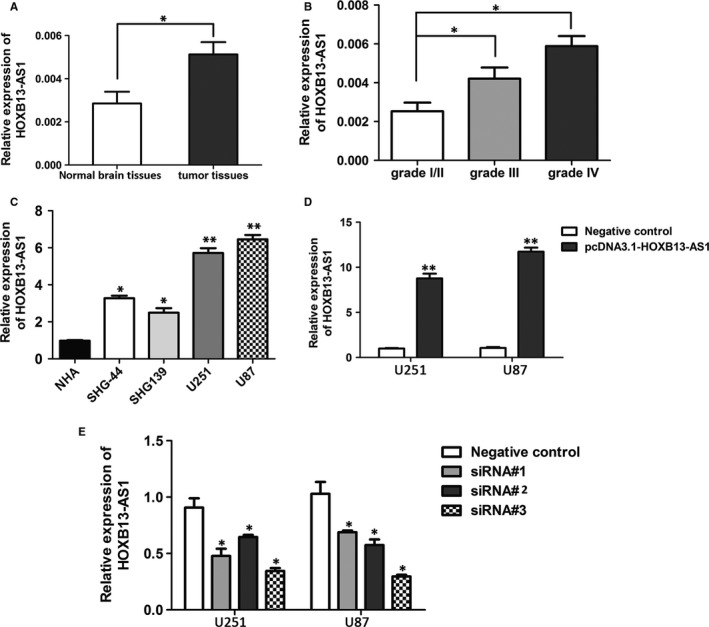
Upregulation of lncRNA HOXB13‐AS1 in glioma tissues and cell lines. A, Relative expression of lncRNA HOXB13‐AS1 in glioma tissues (n = 96) and normal brain tissues (n = 12) was quantified by real‐time PCR (qRT‐PCR). The *P* value was calculated using paired *t* test, *P <* *0.05. B, HOXB13‐AS1 expression showed significant upregulation in high‐grade glioma (III/IV) compared with low‐grade glioma (I+II). Student's *t* test, *P <* *0.05. C, qRT‐PCR analysis of relative expression of HOXB13‐AS1 in glioma cells (U251, U87, SHG‐44, and SHG139) and the normal human astrocytes cell line (NHA), and relative expression of HOXB13‐AS1 were normalized to GAPDH. Data are means ± SD (n = 3). Student's *t* test, **P <* *0.01. D, E, Overexpression and knockdown efficiency of HOXB13‐AS1 after transfection of pCDNA‐HOXB13‐AS1 or siRNAs constructs (siRNA#1, siRNA#2, and siRNA#3) or the negative control in U251 and U87 cells was detected by qRT‐PCR. Data are means ± SD (n = 3). Student's *t* test, *P <* *0.05,**P <* *0.01

In the study cohort, expression level of HOXB13‐AS1 was determined by real‐time PCR in 96 glioma tissue samples with different grades (I, II, III, and IV) and 12 normal brain tissues from individuals who died in traffic accidents. The expression of HOXB13‐AS1 was also upregulated in glioma compared with normal brain tissues (*P *<* *0.05) (Figure 1A). Accordingly, transcript level of HOXB13‐AS1 was markedly higher in glioma tissues with grades III or IV compared with the low‐grade glioma (I+II) (*P *<* *0.05) (Figure 1B). The upregulation of HOXB13‐AS1 in glioma tissues was further verified in glioma cell lines by qRT‐PCR. Consistent with the mRNA expression in glioma tissues, the qRT‐PCR results showed that HOXB13‐AS1 mRNA was significantly upregulated in all tested human glioma cell lines ((U251, U87, SHG‐44, and SHG139), notably U251 and U87), compared with the normal human astrocytes cell line (Figure 1C). This finding implied the prominent function of HOXB13‐AS1 in glioma carcinogenesis.

### 3.2 HOXB13‐AS1–mediate cellular function in glioma

To explore the cellular function of HOXB13‐AS1, we performed the gain or loss‐of‐function assay to study its biologic role in glioma cells. HOXB13‐AS1 overexpression or silence was performed by transfection in glioma cells. The transfection efficiencies were determined through qRT‐PCR (Figure 1D,E). Among the three specific siRNAs (siRNA#1, #2, and #3) targeting different sites of HOXB13‐AS1, the siRNA#3 was chosen for further research because of its stable interfering efficiency in both glioma cell lines.

**Figure 2:**
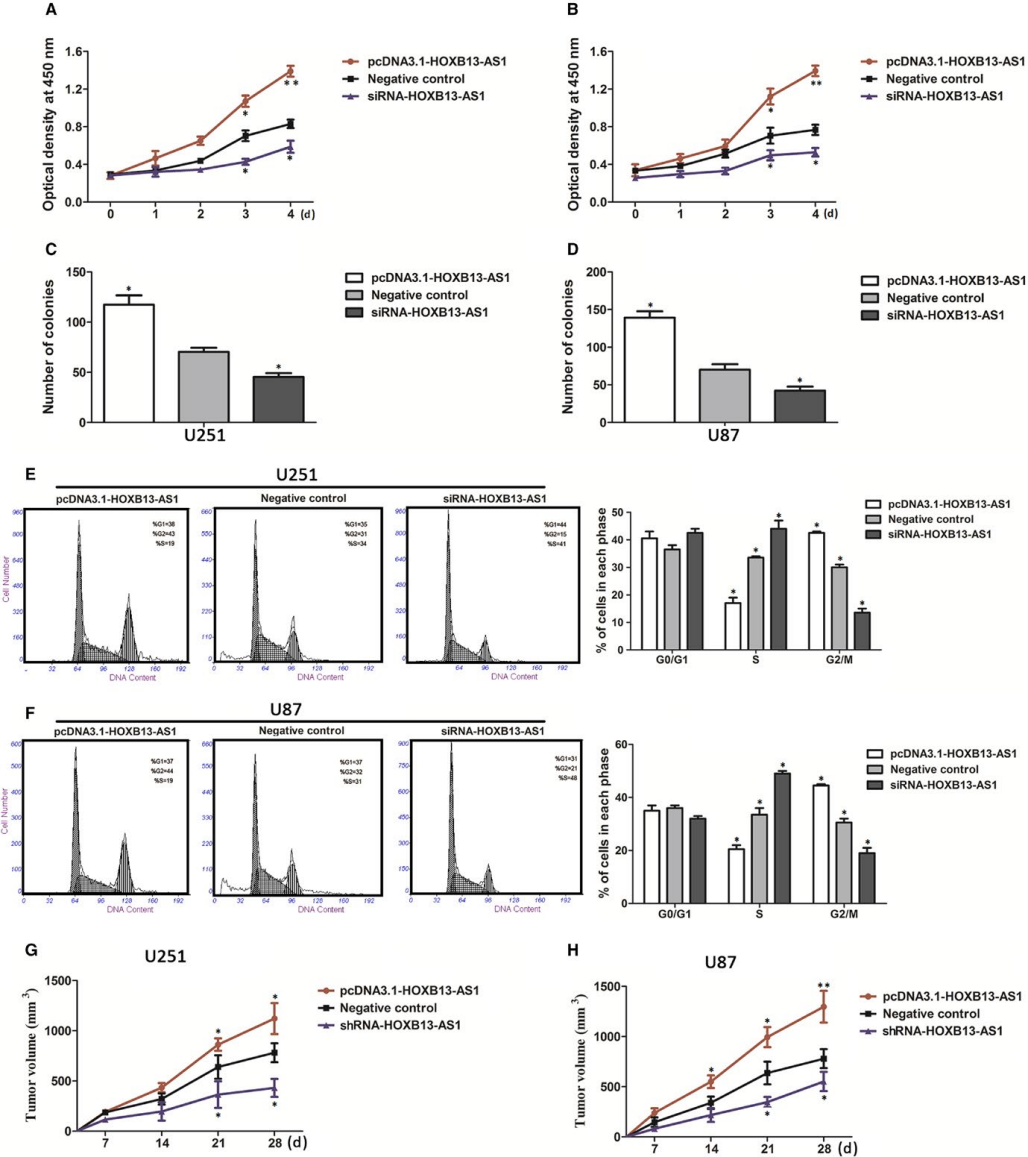
HOXB13‐AS1 increases cell proliferation in glioma cells. A, B, CCK‐8 assay was performed to evaluate the proliferation of U251 and U87 cells transfecting with pcDNA3.1‐HOXB13‐AS1, siRNA‐HOXB13‐AS1, or empty vector. C, D, Effect of HOXB13‐AS1 overexpression or knockdown on colony formation ability of U251 and U87. The data represent the means ± SD from three independent experiments. *P <* *0.05. E, F, HOXB13‐AS1 overexpression promoted cell cycle progression from S phase to G2/M phases, and slicing of HOXB13‐AS1 retarded the S phase to G2/M phases transition. Error bars represent the mean ± SD of three independent experiments, *P <* *0.05. G, H, Effects of overexpression or downexpression of HOXB13‐AS1 on tumor formation ability in vivo. The tumor size was much larger in HOXB13‐AS1‐overexpression subcutaneous mice compared with those in control group, while tumor size of subcutaneous mice in the HOXB13‐AS1‐downexpression groups was smaller than those in control groups. Data were mean ± SD (N = 8). The *P* values were calculated using ANOVA, *P <* *0.05,**P <* *0.01. N, number

Next, we investigated the effects of HOXB13‐AS1 overexpression or knockdown on cell proliferation, colony formation, and cell cycle distribution. The CCK‐8 assay showed that the HOXB13‐AS1 overexpression significantly increased the cell proliferation in both U251 (Figure 2A) and U87 cells (Figure 2B) (U251 cells: 58.3% increase, U87 cells: 60.2% increase) on day 4, while silence of HOXB13‐AS1 expression inhibited cell growth (U251 cells: 24.6% decrease, U87 cells: 21.4% decrease), compared with control cells. These results were confirmed by colony formation assays (Figure 2C,D). Further, flow cytometry was conducted to analyze the role of HOXB13‐AS1 on cell cycle in glioma cells, and results indicated that increase in HOXB13‐AS1 expression promoted cell cycle progression from S phase to G2/M phase in comparison with control cells, and knockdown of HOXB13‐AS1 retarded the S phase to G2/M phase transition (Figure 2E,F).

### 3.3 HOXB13‐AS1 promotes glioma cell proliferation in vivo

To determine whether HOXB13‐AS1 affects tumor proliferation in vivo, glioma cells infected with pCDNA‐HOXB13‐AS1, sh‐HOXB13‐AS1, or negative control (empty vector) were injected into male nude mice. Consistent with the result in cultured tumor cells, tumor growth of subcutaneous xenograft tumors in the HOXB13‐AS1‐overexpression groups was dramatically increased when compared to the empty vector group (1121.6 ± 46.0 mm^3^ vs 781.5 ± 31.7 mm^3^ of U251 cells, *P *<* *0.05; 1197.6 ± 45.2 mm^3^ vs 810.5 ± 33.1 mm^3^ of U87 cells, *P *<* *0.05; Figure 2G,H), while the downregulation of HOXB13‐AS1 expression in the sh‐HOXB13‐AS1 group resulted in significant decrease in growth (431.4 ± 36.2 mm^3^ vs 781.5 ± 31.7 mm^3^ of U251 cells, *P *<* *0.01; 552.4 ± 34.2 mm^3^ vs 810.5 ± 33.1 mm^3^ of U87 cells, *P *<* *0.05; Figure 2G,H). These data indicate that overexpression of HOXB13‐AS1 could increase glioma cell proliferation.

### 3.4 HOXB13‐AS1 epigenetically regulate neighboring gene HOXB13 transcription by binding to EZH2

**Figure 3:**
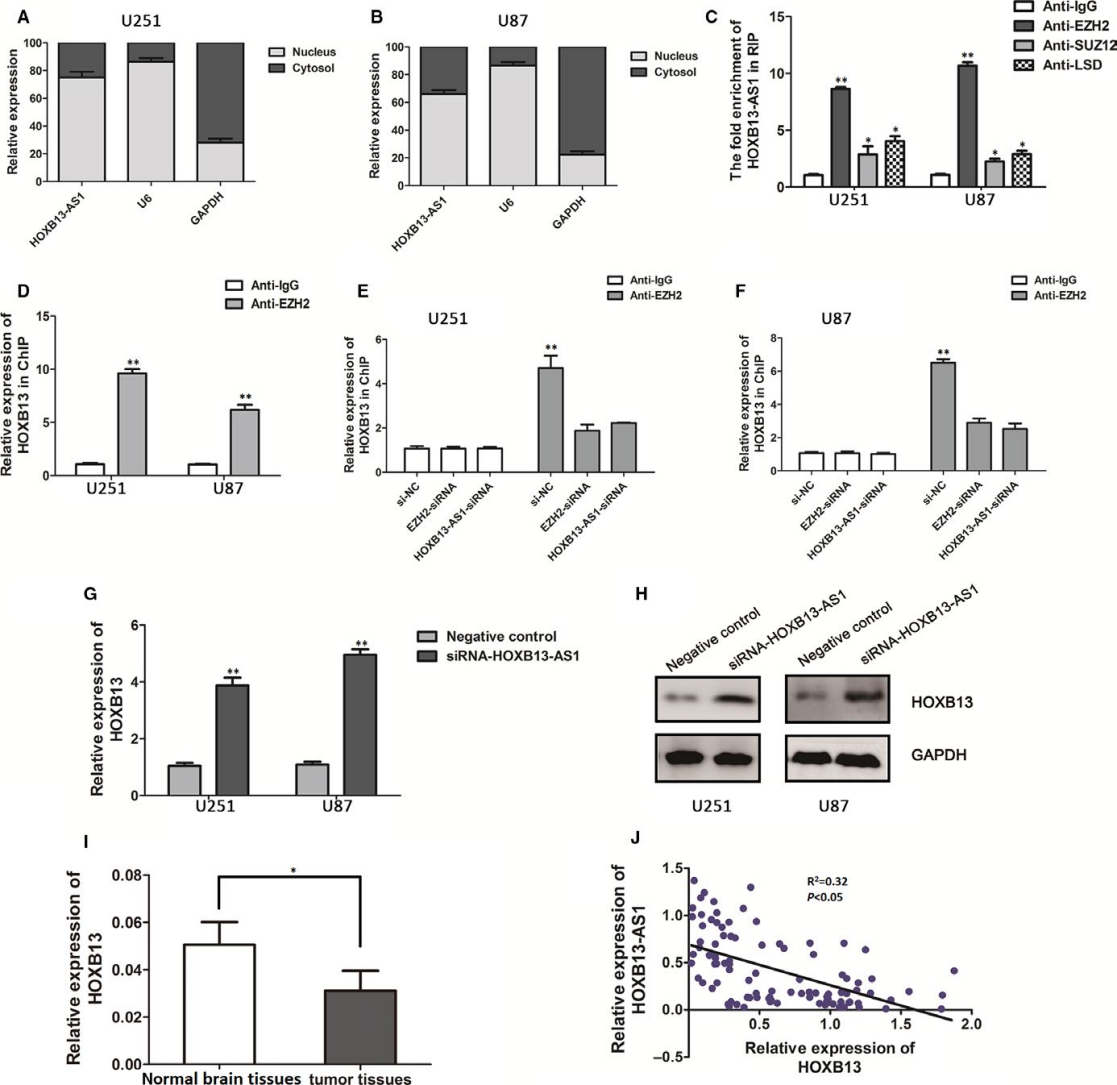
HOXB13‐AS1 epigenetically regulate HOXB13 gene transcription by through interacting with EZH2. A, B, Distribution of HOXB13‐AS1 in U251 and U87 cells was determined by qRT‐PCR. HOXB13‐AS1 was mainly localized in nucleus of U251 and U87 cells (mean ± SD). C, RIP assay was performed to determine the association between HOXB13‐AS1 and catalytic components (EZH2, SUZ12, and LSD1) of PRC2. The fold enrichment of HOXB13‐AS1 in U251 and U87 cells with antibodies against EZH2, SUZ12, and LSD1 is relative to nonspecific IgG control. Error bars represent the mean ± SD of three independent experiments, Student's *t* test, *P <* *0.05. D, ChIP‐PCR assays were performed to determine the interaction between the promoter region of HOXB13 and EZH2 with antibodies against EZH2 and IgG (mean ± SD), Student's *t* test, *P <* *0.05. E, F, ChIP‐PCR assays of EZH2 enrichment of the promoter region of HOXB13 after the silence of EZH2 or HOXB13‐AS1 in U251 and U87 cells with antibodies against EZH2. HOXB13‐AS1 affects the mRNA levels and protein levels of HOXB13 in U251 and U87 cells after transfection of the siRNA of HOXB13‐AS1 as detected by qRT‐PCR assay (G) and Western blot assay (H). Data are indicated as mean ± SD for three independent experiments. Student's *t* test, *P <* *0.05, **P <* *0.01. I, qRT‐PCR analysis of relative expression of HOXB13 in glioma tissues compared with normal brain tissues. HOXB13 expression levels were normalized to GAPDH. The *P* value was calculated using paired *t* test, *P <* *0.05. J, The association between the HOXB13‐AS1 and HOXB13 mRNA expression levels was determined by the Pearson's correlation analysis (*R* ^2 ^= 0.32, *P *<* *0.05)

Accumulated evidence has indicated that typical lncRNAs often display much more complicated regulatory effects on targeted genes expression by binding to various catalytic components (EZH2, SUZ12, and LSD1) of polycomb repressive complex 2 (PRC2),^35^, ^36^, ^37^ which can catalyze the di‐ and trimethylation of lysine residue 27 of histone 3 (H3K27me3). To investigate the potential mechanism and downstream targets of HOXB13‐AS1‐mediated regulation in glioma cells. Subcellular fractionation location assays firstly demonstrated that the localization of HOXB13‐AS1 was mainly distributed in the nucleus of U251 and U87 cells (Figure 3A,B), suggesting HOXB13‐AS1 could regulate underlying targets expression at transcriptional level. Then, we employed RIP assay to assess the binding activity between HOXB13‐AS1 and the core catalytic components (EZH2, SUZ12, and LSD1) of PRC2 in U251 and U87 cells. The result detected by qRT‐PCR showed an 8.4‐fold and 11.3‐fold enrichment of HOXB13‐AS1 in U251 and U87 cells with an antibody against EZH2 as compared with the nonspecific IgG control (Figure 3C); however, there was a slight enrichment of HOXB13‐AS1 with an antibody against SUZ12 or LSD1.

Intriguingly, HOXB13‐AS1 neighbors the HOXB13 gene. Given the positional effect of HOXB13‐AS1 and HOXB13 gene in the same regions, we hypothesis that HOXB13‐AS1 may modulate its neighboring gene HOXB13 expression through recruiting EZH2 to the HOXB13 gene promoter. Firstly, using UCSC Genome Bioinformatics Site, high enrichment of H3K27me3 at the promoter of HOXB13 was found. We further confirm the association of EZH2 with HOXB13 gene promoter by performing ChIP assays in glioma cells. The data presented in Figure 3D showed a 9.3‐fold and 6.1‐fold enrichment of EZH2 in HOXB13 promoter as detected by qRT‐PCR compared with control in U251 and U87 cells. The enrichment ability of EZH2 was significantly decreased after knockdown of EZH2 by siRNA (Figure 3E,F), supporting the idea that HOXB13 expression was regulated by EZH2.

We further explored whether HOXB13‐AS1 is required for occupation of EZH2 at chromatin region or not. As expected, depletion of HOXB13‐AS1 (siRNA) decreased binding of EZH2 in the region of HOXB13 promoter compared to cells transfected with si‐NC (Figure 3E,F). In order to confirm the above results of CHIP–PCR, qRT‐PCR was used to detect HOXB13 expression level after manipulating HOXB13‐AS1 expression in glioma cells. We observed a significant upregulation of the HOXB13 mRNA level and protein level in the HOXB13‐AS1‐downregulated cells (Figure 3G,H). These results suggest that HOXB13‐AS1 negatively regulated HOXB13 expression by recruiting EZH2 to HOXB13 promoter region. In an attempt to understand the potential role of HOXB13 to glioma development, qRT‐PCR analysis found that HOXB13 expression was significantly decreased in 96 glioma tissues, compared to normal brain tissues (*P *<* *0.05, Figure 3I). Furthermore, HOXB13‐AS1 expression was negatively correlated with HOXB13 levels (*R* ^2 ^= 0.32, *P *<* *0.05; Figure 3J), confirming that HOXB13 was a downtarget of HOXB13‐AS1‐regulated genes.

### 3.5 EZH2 recruited DNMT3B to HOXB13 promoter and regulate HOXB13 methylation

**Figure 4:**
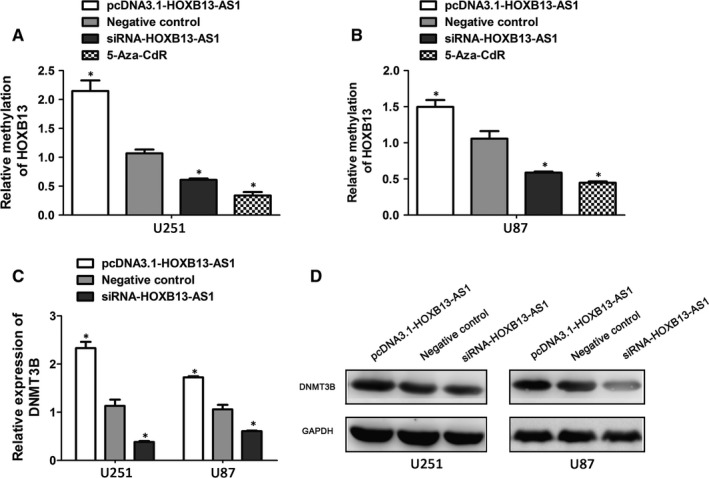
EZH2 recruited DNMT3B to HOXB13 promoter and regulate HOXB13 methylation. A, B, The bisulfite sequencing PCR analysis (BSP) of the methylation levels of HOXB13 in 5‐Aza‐CdR‐treated or HOXB13‐AS1‐downregulated glioma cells. Results are mean ± SD, *P <* *0.05. Quantification of DNMT3B at mRNA expression levels and protein levels after the overexpression or silence of HOXB13‐AS1 in U251 and U87 cells by qRT‐PCR assay (C) and Western blot assay (D). Error bars indicate mean ± SD, *P <* *0.05

Epigenetic mechanisms, such as DNA methylation, play a crucial role in mammalian development and maintained by specific proteins, DNA (cytosine‐5) methyltransferases (DNMTs). The aberrant DNA methylation patterns in gene promoter are linked to a growing number of diseases, including cancer.^38^, ^39^ Recent studies have identified that HOXB13, a direct DNMT3B target gene, is hypermethylation at the upstream CpG island and functions as a tumor suppressor expression in the colon cancer and renal cell carcinoma.^27^, ^40^ In this study, by performing BSP assay, we found that the methylation level of HOXB13 promoter region was higher in HOXB13‐AS1‐upregulated cells compared to NC cells (Figure 4A,B). Meanwhile, the methylation levels of HOXB13 decreased after treatment with DNA methyltransferase inhibitor 5‐Aza‐2‐deoxycytidine (5‐Aza‐CdR; Sigma‐Aldrich) or in HOXB13‐AS1‐downregulated cells (Figure 4A,B). These result indicated that the methylation level of HOXB13 is consistent with HOXB13‐AS1 expression. Next, to better characterize the way by which HOXB13‐AS1 regulated the HOXB13 methylation, we measured the expression of DNMTs (DNMT1, DNMT3A, and DNMT3B), which is recruited to gene promoter region by EZH2 and leading to the methylation of H3K27. Notably, only DNMT3B expression decreased significantly in HOXB13‐AS1‐downregulated cells (Figure 4C,D). In contrast, HOXB13‐AS1 overexpression increased mRNA and protein levels of DNMT3B (Figure 4C,D). These findings suggested that upregulation of HOXB13‐AS1 methylated adjacent gene HOXB13 by increasing expression of DNMT3B.

## 4 DISCUSSION

lncRNAs, a large subgroup of the noncoding transcripts, recently have been identified to play crucial roles in cellular differentiation, development, and disease progression, especially in several kinds of tumors. Dysregulation of lncRNAs has been implicated in glioma. In our present study, we found that a newly identified lncRNA HOXB13‐AS1 is aberrantly expressed in glioma tissues and promotes cell growth by affecting cell cycle progression, suggesting that HOXB13‐AS1 might be a novel biomarker for glioma carcinogenesis.

**Figure 5:**
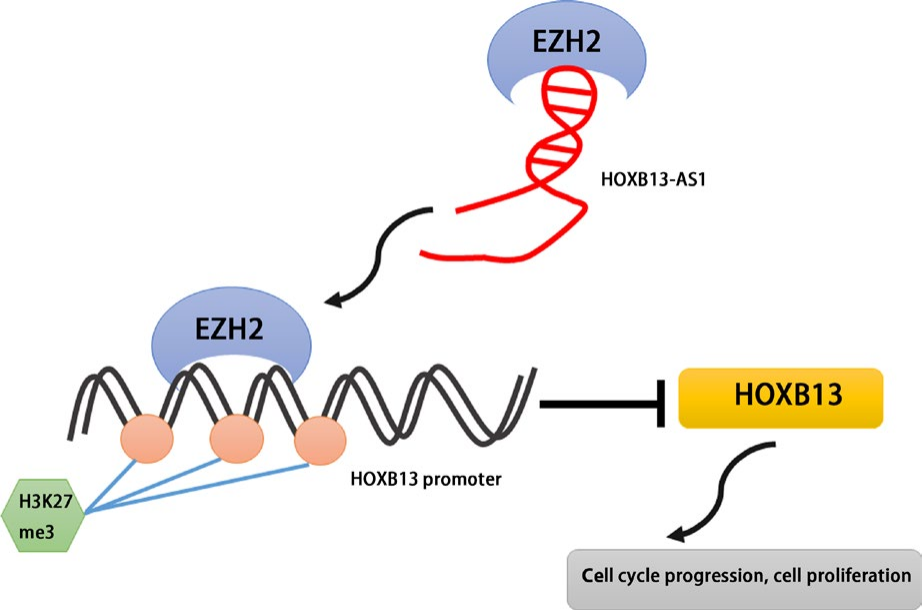
Flow diagram of the mechanism of HOXB13‐AS1 on glioma

The function and underlying molecular mechanisms of lncRNAs in carcinogenesis have led to an intense focus. Many lncRNA has been reported to control diverse biological processes by multiple mechanisms, including modulating transcription, epigenetic modification, and as competing endogenous RNAs (ceRNAs).^41^, ^42^, ^43^, ^44^ LncRNAs can function as a miRNA sponge to regulate gene activation or repression. Another mechanism of action by which lncRNAs work is via base‐pairing interactions to degrade the mRNA. Importantly, an emerging view of lncRNA is that they can recruit the component of chromatin‐modifying complexes (PRC2) to the specific target genes to achieve transcriptional regulation. In the current study, the biological functions and underlying mechanism of HOXB13‐AS1 upregulation are largely unknown in glioma development. We report here, for the first time, that transcript level of HOXB13‐AS1 is significantly upregulated in glioma tissues and cells, and HOXB13‐AS1 expression level was negatively associated with the mRNA level of its neighbor gene HOXB13 in glioma specimens. Functional assay in vitro and in vivo showed that HOXB13‐AS1 ectopic expression promoted cell proliferation possibly via accelerating cell cycle progression, while silence of HOXB13‐AS1 in glioma cells leads to suppression of tumor growth. To identify the underlying mechanism of HOXB13‐AS1 suppressing HOXB13 expression, we performed comprehensive studies. Firstly, we examined the distribution of HOXB13‐AS1 in glioma cells. We showed that HOXB13‐AS1 was mainly located in the nucleus, implying that they seem to predominantly action biological functions at transcriptional level. EZH2, as a component of the PRC2 complex and an epigenetic gene silencer, can repress gene transcription through trimethylation of lysine 27 on histone H3 (H3K27) across its promoter. EZH2 has been functionally associated with human diseases.^45^, ^46^, ^47^ Given that accumulating studies have demonstrated the interact between lncRNAs and EZH2, we hypothesize that HOXB13‐AS1 might regulate expression of downstream gene HOXB13 via EZH2‐driven H3K27 methylation. Indeed, we found the binding activity between HOXB13‐AS1 and the core catalytic component (EZH2) of PRC2 in U251 and U87 cells by RIP experiment. In addition, using UCSC Genome Bioinformatics Site, we found high enrichment of H3K27me3 at the promoter of HOXB13. We further confirm the association of EZH2 with HOXB13 gene promoter by performing ChIP assays in glioma cells. The binding affinity of EZH2 ability to HOXB13 promoter was significantly decreased after knockdown of EZH2 by siRNA, supporting the idea that HOXB13 expression was regulated by EZH2. As expected, we confirmed that knockdown of HOXB13‐AS1 decreased binding affinity of EZH2 to the region of HOXB13 promoter. These results support that HOXB13‐AS1 can epigenetically regulate neighboring gene HOXB13 transcription by binding to EZH2 (Figure 5).

In mammalian cells, gene methylation patterns are determined by the joint action of three specific proteins, DNA methyltransferases (DNMT1, DNMT3A, and DNMT3B), play important role in regulation of numerous biological processes. Aberrant methylation can cause developmental defects and complex diseases, including malignancies. Multiple lines of evidence have uncovered large subgroups of lncRNAs as new important players in DNA methylation regulation of adjacent gene by recruiting chromatin‐modifying complexes to affect chromatin modifications.^34^, ^42^ The developmentally regulated H19 has been shown to interact with the S‐adenosylhomocysteine hydrolase (SAHH), altering DNMT3B‐mediated methylation of Igf2‐H19‐Nctc1 locus.^48^ To determine whether HOXB13‐AS1 knockdown could alter its nuclear interaction with EZH2 and modulate transcription by affecting gene methylation, the methylation level of HOXB13 promoter region was investigated in cells with HOXB13‐AS1‐altered or treatment of DNA methyltransferase inhibitor. HOXB13‐AS1 upregulation increased the methylation levels of HOXB13 methylation, while HOXB13‐AS1‐downregulation or DNA methyltransferase inhibitor decreased the methylation levels of HOXB13. One study has shown that HOXB13 as a direct DNMT3B target is aberrantly methylated at an upstream CpG island, and functions’ tumor suppressor properties in colorectal cancer cells. To further address whether DNMT3B is recruited to HOXB13 gene promoter region by EZH2 and leading to the methylation of H3K27, we measured the expression of DNMT3B in HOXB13‐AS1‐altered cells. The activity of DNMT3B might be subjected to regulation by HOXB13‐AS1‐mediated mechanisms. All these findings indicated that HOXB13‐AS1 regulated DNA methylation of surround gene HOXB13 by increasing expression of DNMT3B.

In summary, this study has identified an epigenetic player, HOXB13‐AS1, which was overexpressed in the high‐grade gliomas compared with low‐grade gliomas and normal brain tissues, and upregulation of HOXB13‐AS1 enhanced cell growth and promoted cell cycle progression. The molecular mechanisms analysis revealed that HOXB13‐AS1 could epigenetically silenced neighbor gene HOXB13 by EZH2‐dependent H3K27me3 modification. LncRNA HOXB13‐AS1 might be considered as the potential predictor for glioma diagnosis.

## CONFLICT OF INTEREST

The authors declare no competing financial interests.
